# Chasing coevolutionary signals in intrinsically disordered proteins complexes

**DOI:** 10.1038/s41598-020-74791-6

**Published:** 2020-10-21

**Authors:** Javier A. Iserte, Tamas Lazar, Silvio C. E. Tosatto, Peter Tompa, Cristina Marino-Buslje

**Affiliations:** 1 Fundación Instituto Leloir, Patricias Argentinas 435, Buenos Aires, Argentina; 2 VIB-VUB Center for Structural Biology, Vrije Universiteit Brussel, Pleinlaan 2, 1050 Brussels, Belgium; 3 Structural Biology Brussels, Department of Bio-Engineering, Vrije Universiteit Brussel, Pleinlaan 2, 1050 Brussels, Belgium; 4 Department of Biomedical Sciences, University of Padova, 35121 Padova, Italy; 5 Institute of Enzymology, Research Centre for Natural Sciences, Hungarian Academy of Sciences, Magyar Tudósok Körútja, Budapest, Hungary

**Keywords:** Computational biology and bioinformatics, Structural biology

## Abstract

Intrinsically disordered proteins/regions (IDPs/IDRs) are crucial components of the cell, they are highly abundant and participate ubiquitously in a wide range of biological functions, such as regulatory processes and cell signaling. Many of their important functions rely on protein interactions, by which they trigger or modulate different pathways. Sequence covariation, a powerful tool for protein contact prediction, has been applied successfully to predict protein structure and to identify protein–protein interactions mostly of globular proteins. IDPs/IDRs also mediate a plethora of protein–protein interactions, highlighting the importance of addressing sequence covariation-based inter-protein contact prediction of this class of proteins. Despite their importance, a systematic approach to analyze the covariation phenomena of intrinsically disordered proteins and their complexes is still missing. Here we carry out a comprehensive critical assessment of coevolution-based contact prediction in IDP/IDR complexes and detail the challenges and possible limitations that emerge from their analysis. We found that the coevolutionary signal is faint in most of the complexes of disordered proteins but positively correlates with the interface size and binding affinity between partners. In addition, we discuss the state-of-art methodology by biological interpretation of the results, formulate evaluation guidelines and suggest future directions of development to the field.

## Introduction

Many positions in protein sequences do not evolve independently from each other, but rather bear a pattern of inter-relationship since some changes are accepted in evolution only if there is a compensatory change somewhere else in the protein which ensures that structure and/or function are maintained. The correlation pattern between positions appears as the result of a process of concurrent mutations and it is generally known as “coevolution”. Coevolving residues are usually in spatial proximity to each other, therefore analyzing the correlated residue variation in a protein family became a common technique for contact prediction in globular domains^1–9^. Structure prediction tools showed an improved performance in the Critical Assessment of Structure Prediction (CASP) experiment upon the incorporation of coevolutionary information^10^. More recently, evolutionary couplings were observed across protein–protein interfaces, mostly in prokaryotic complexes^1,11–13^.

The bottleneck of a covariation analysis is usually the quality and size of the multiple sequence alignment (MSA) used. The sensitivity of methods depends on the number and diversity of sequences in respective MSAs, and each method has its own requirements to achieve meaningful predictive performances^14–17^.

For a great number of globular proteins it is possible to generate such alignments, but it is very difficult for intrinsically disordered proteins (IDPs) and proteins with intrinsically disordered regions (IDRs). Coevolutionary information in IDP complexes has not been systematically analyzed because of the technical difficulty to obtain reliable results, e.g. to generate a well-aligned MSA from a large number of divergent homologs since standard tools have been optimized for globular proteins.

The estimated fraction of disordered proteins in eukaryotic proteomes is over 40%^18–20^, and these proteins mediate a plethora of protein–protein interactions^21^, highlighting the importance of addressing their sequence covariation-based inter-protein contact prediction.

Analysis of coevolved inter-protein covarying residue pairs is possible if the paired MSA containing paired orthologs has an effective size, and the coevolutionary signal is strong enough. Clearly, this requirement makes the analysis of disordered protein complexes much more challenging and restricted. Up till now, the use of inter-protein covariation signal has only been demonstrated to be effective in modeling the complexes of globular proteins, as demonstrated in the Critical Assessment of Prediction of Interactions (CAPRI) experiment^22,23^.

In the present work, we perform an analysis of two databases of IDP complexes: the database of intrinsically disordered binding sites (DIBS)^24^ and the mutual folding induced upon binding database (MFIB)^25^. We analyzed 287 complexes involving disordered proteins or regions that fall into two flavors: (1) complexes between a disordered protein and a globular domain and (2) complexes where the two chains are disordered on their own and become structured upon binding.

Through these analyses, we established that coevolutionary signals between interacting proteins are generally faint but positively correlate with the affinity of binding. By way of biological interpretation of the results, we also thoroughly discuss the challenges and possible limitations of the analysis, and formulate guidelines to the community on how to carry out the critical evaluation of the underlying methods. In essence, this analysis opens novel avenues of understanding protein–protein interactions and suggests future directions of development to the field.

## Results

### Intra- and inter-protein (complexes) covariation

The three-dimensional structure imposes strong constraints on amino acid replacement during the course of evolution. Phylogenetically related positions evolve in a coordinated manner, leaving a recognizable footprint in sequence alignments. By analyzing the sequence variability within a homologous protein family, it is possible to infer the coevolution between positions (columns in an MSA) from their covariation.

In this work, we evaluated the coevolution between IDPs (or IDRs) and their partners in protein complexes. To this end, we analyzed two databases that present data on disordered regions in complexes: DIBS that contains complexes between an ordered and a disordered partner, and MFIB that contains complexes between two IDRs that undergo mutual folding when they bind to each other.

It is assumed that the vast majority of coevolving pairs are in contact with each other, so a reasonable approach to compare the performance of different methods is to measure their capability of predicting residue contacts.

To distinguish true coevolutionary couplings from the noise of covariations, we tested a naive predictor based solely on residue distances in the sequence, without the use of evolutionary (i.e. MSA-based) or structural information. The results of the naive predictor suggest the importance of filtering out at least five neighboring residues to have an unbiased evaluation of the performance of contact prediction (i, i + 5) (Supplementary Figure S1). With our definition of five residues as a threshold to evaluate coevolution, we might lose some truly coevolving positions, but on the other hand, we also lower the number of trivial hits and false positives, suppressing non-phylogenetically related patterns.

Other covariation methods consider all contacts between residues, or only exclude contacts between covalently bound residues, and/or contacts between residues i and i + 4^5,15,26^. CASP experiments suggest that considering contacts between residues within six positions in the sequence might significantly bias covariation analysis^27^.

**Figure 1:**
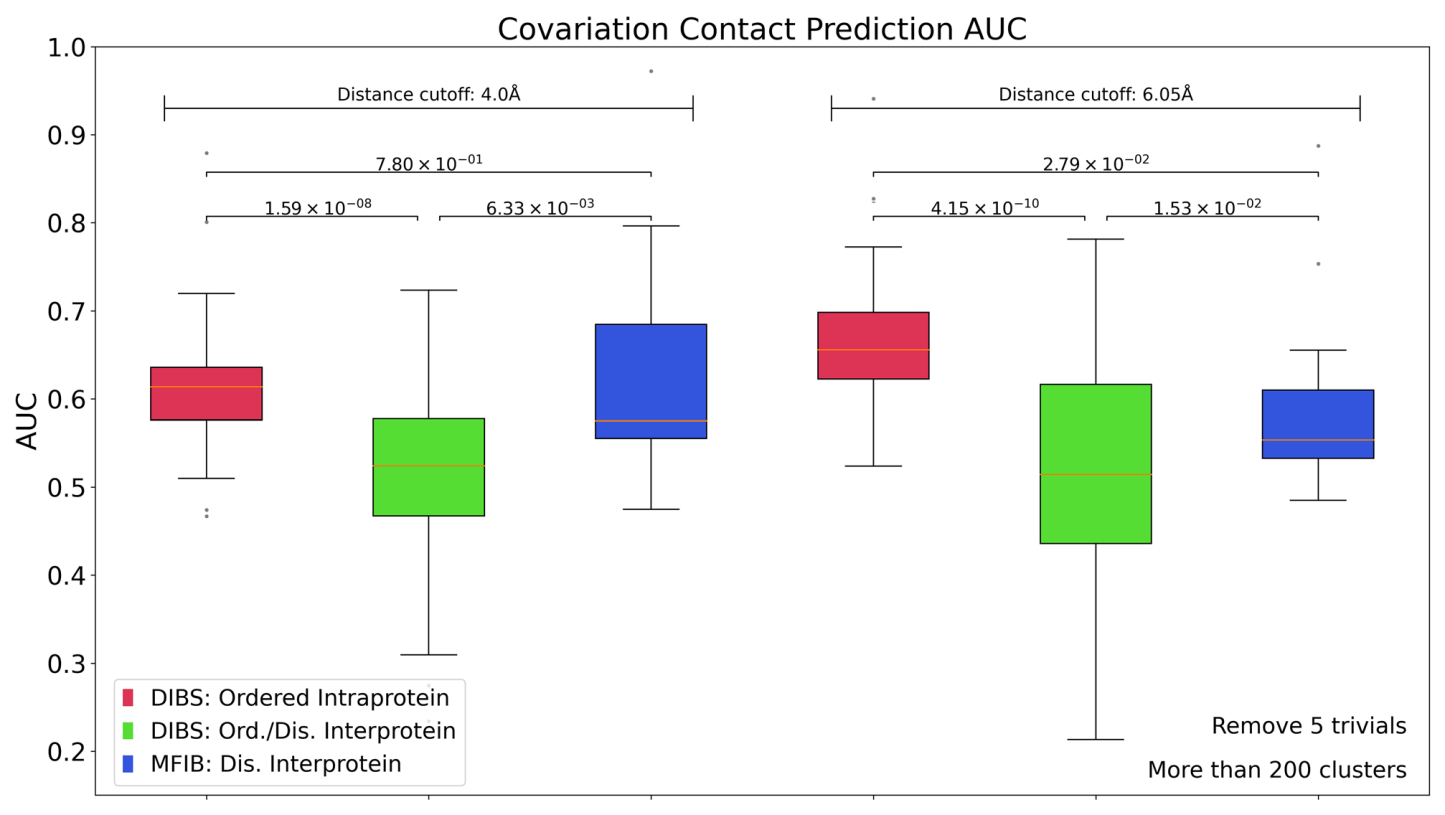
AUC for contact prediction. Red: intra-molecular ordered (globular) partner in DIBS database; green: inter-molecular contacts of DIBS database complexes; blue inter-molecular contacts of MFIB database complexes. Results shown with MSAs having more than 200 sequence clusters, 6.05 and 4 Å distance between heavy-atom pairs to define a contact, excluding five neighbor residue contacts (as trivial ones). Two-sample T-test for unequal variance was performed and the p-values are shown.

We use the intra-molecular coevolution values of the ordered globular protein partners of IDPs/IDRs in the DIBS database as a reference to compare with the inter-molecular values between interacting proteins. Supplementary Figure S2 shows the comparison between coevolution performance for intra-protein (ordered partner in DIBS) and inter-protein contact prediction (between ordered and disordered proteins in DIBS dataset) under several conditions (including the number of sequence clusters, neighboring residues to be considered as trivial and 3D distance definition). For clarity, we show in Fig. 1 the area under the ROC curve (AUC) for contact prediction (as a proxy to coevolution) using MSAs containing at least 200 clusters at 80% sequence identity, here, we consider the contacts between neighboring five residues as trivial and define those residues in “contact”, if they have any of their heavy atoms closer than 4 and 6.05 Å in space (see other conditions in Supplementary Figure S2). We found that in all conditions, the AUC for inter-protein coevolution prediction is lower than the intra-protein coevolution values (within the ordered partner). The low predictability of inter-protein coevolution in DIBS complexes shows that these proteins do not covary to the extent observed for complexes of ordered bacterial proteins^11,28^ or intra-protein residues.

**Figure 2:**
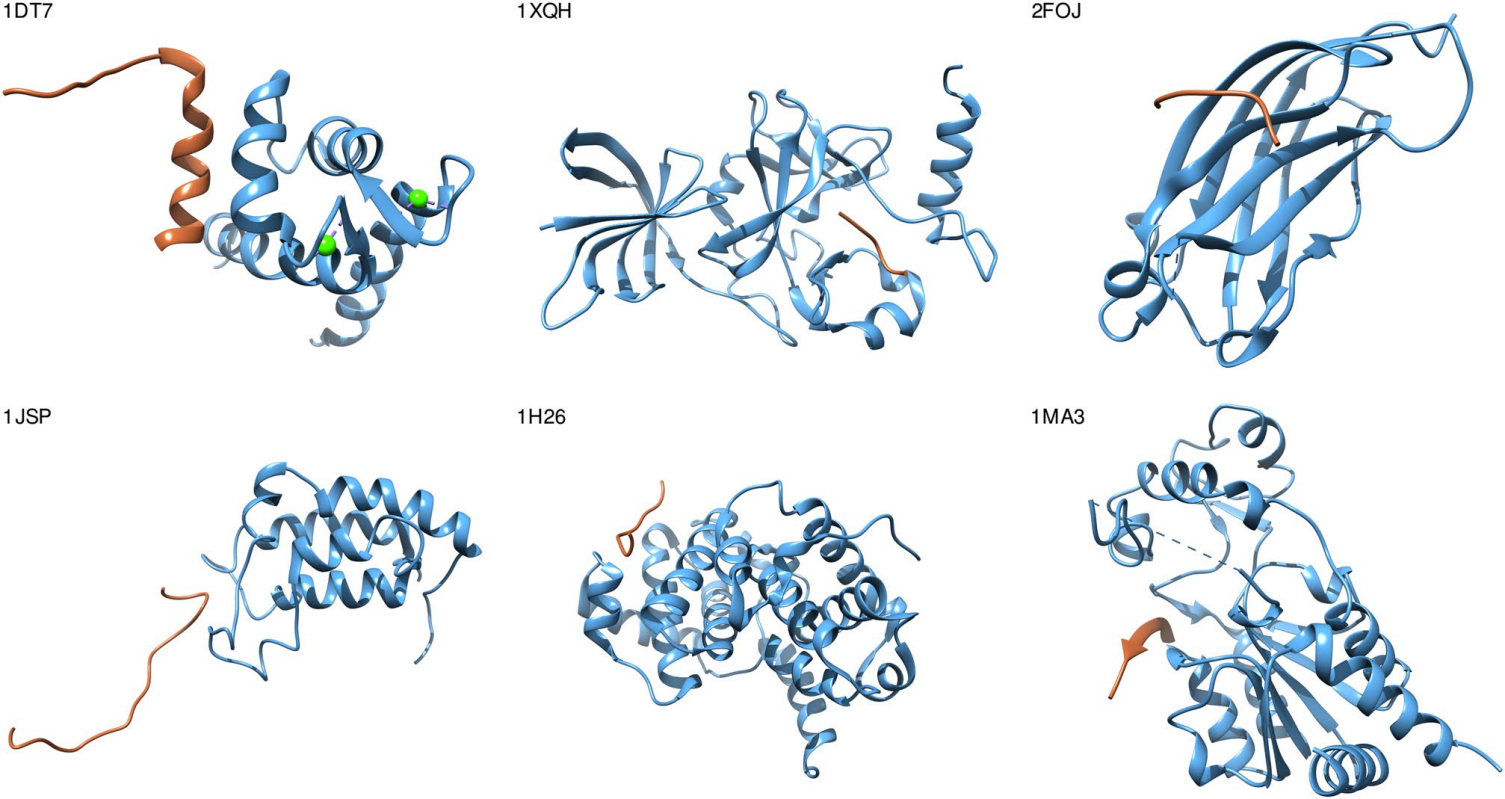
Ribbon representation of p53 residues: region 358–388 (regulatory domain) interacting with many non-homologous proteins that have different folds. Color brown, P53 region; light blue, binding partners (PDB codes: 1dt7; 1xqh; 2foj; 1h26; 1jsp and 1ma3). The image was made using Chimera 1.14.1^36^.

One of the multiple causes behind this phenomenon may be the promiscuity of disordered proteins^29^. As numerous IDRs can bind different partners with adjacent or even overlapping surfaces (cf p53^30^), it is paradoxical that they could possibly coevolve with all different binding partners at the same time. As an example, Fig. 2 shows the structures of different complexes of p53 residues 358–388 (regulatory domain) interacting with several non-homologous proteins of different folds and different surface properties. It can be seen that the same p53 IDR adopts different conformations depending on its partner.

Another factor could be that globular proteins and IDRs do not evolve at the same rate. IDRs are known for their rapid evolution^31^ which is faster than that of their ordered counterparts. Another consideration is the fuzziness of the IDP complexes^32^. It is widely accepted that IDPs exist as dynamic ensembles of conformations, rather than having a single conformation^33^, hence the structures evaluated might not represent the dominant or only conformation the proteins adopt in their complexes. In case of X-ray complexes, crystal packing might pose some artificial constraints on the complex, while both NMR and X-ray structures may have been measured under conditions far from being physiological. This can also lead to the overestimation of the stability of the complex, i.e. the affinity of binding. Many of these complexes may arise from transient, short-lived and unstable interactions, not strong enough to leave a covariation signal. Supplementary Figure S3 shows results with a complete set of different variable conditions, all pointing to the same results. In Supplementary Figure S3, we included the protein contacts within the disordered part, even though we do not consider this data relevant as IDRs have very few intra-protein contacts and even less when neighboring residues (trivial contacts) are removed from the analysis.

Another type of IDP/IDR complex is represented by structures deposited in the MFIB database. This database contains the structures of complexes that form upon the binding of two disordered partners to each other. We performed a two-sample t-test of means with unequal variances to compare the relevant AUC values. The statistics of analysis showed that the mean AUC of inter-protein contact prediction in the MFIB database is higher than the inter-protein AUC mean in the DIBS database, and is closer to the performance of intra-molecular contact prediction on globular proteins (ordered partner of DIBS), although the number of analyzed MFIB complexes is small (Fig. 1). Supplementary Figure S4 shows the results of applying different conditions to the analysis of MFIB complexes (4 and 6.05 Å distance, 0, > 100 and > 200 number of clusters at 62 and 80% clustering threshold).

This result agrees with our observation that the three-dimensional structure of MFIB complexes are often similar to proper folds of globular proteins. In fact, some complex structures (IDR and globular partner together) belong to known fold families of monomeric ordered proteins (see examples in Supplementary Figure S5).

### Inter-protein coevolution correlates with affinity between partners

**Figure 3:**
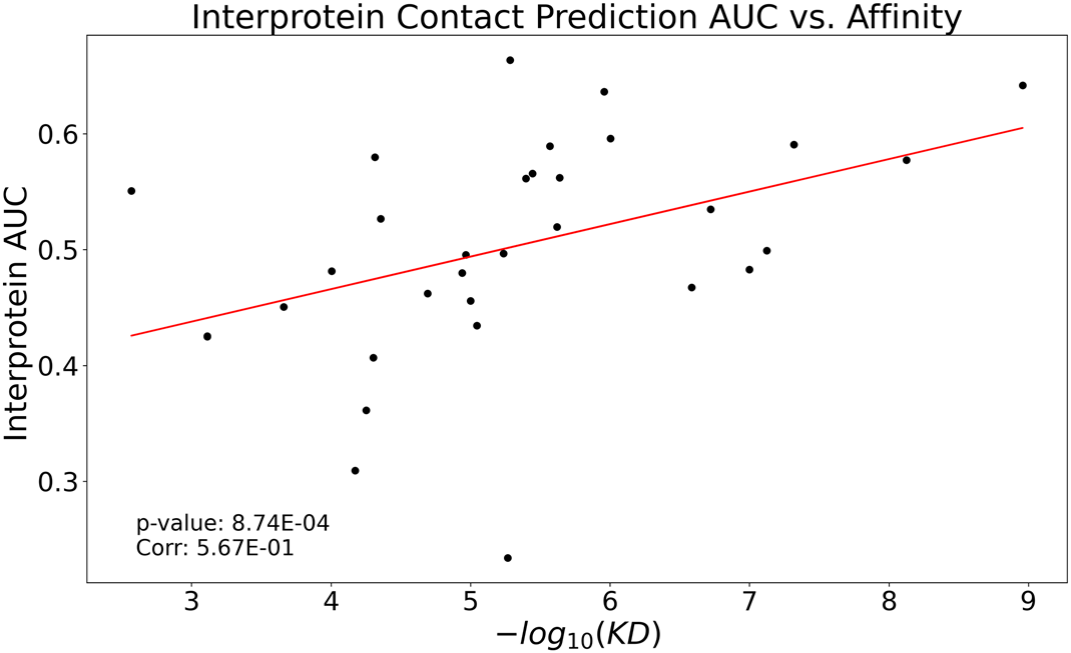
Correlation between Kd and AUC of inter-protein contact prediction. We only considered protein pairs from DIBS heterodimers (rho = 0.567, pval = 8.7 × 10^–4^).

We found a significant Spearman correlation of 0.567 between affinity, measured as the dissociation constant (Kd) of an IDR interacting with a single globular protein, and inter-protein coevolution, for complexes in the DIBS database. Figure 3 shows the correlation between Kd and the AUC for inter-protein contact prediction in MSAs having more than 200 clusters (Complexes having more than 100 clusters have an even better p-value of 7.15 × 10^–4^, and a correlation of 0.467 (Supplementary Figure S6a). This goes in line with the results of previous studies that describe higher coevolution signals between proteins in permanent complexes than in transient ones^34,35^. Kd values were taken from the database (DIBS) as it also stores experimentally determined affinities measured by a diverse set of techniques (including tryptophan fluorescence assay, fluorescence polarization anisotropy, surface plasmon resonance spectroscopy, isothermal titration calorimetry, and NMR spectra analysis, among others). We should not miss, though, that comparing Kd values from different sources may introduce additional noise into the analysis, i.e. the results presented here should be taken with care. Nonetheless, we also found correlations between the area of the interface (interaction surface) between the partners and both the AUC and Kd values (Supplementary Figure S6b). The area of the interface was estimated as the solvent-excluded surface and computed using Chimera 1.14.1^36^.

This result suggests that complexes between IDPs/IDRs and globular partners with higher binding affinity (lower Kd) might have coevolved more recognizably on the residue level, and an evolutionary footprint can be better seen in their sequences as covariation between pairs of interface residues.

**Figure 4:**
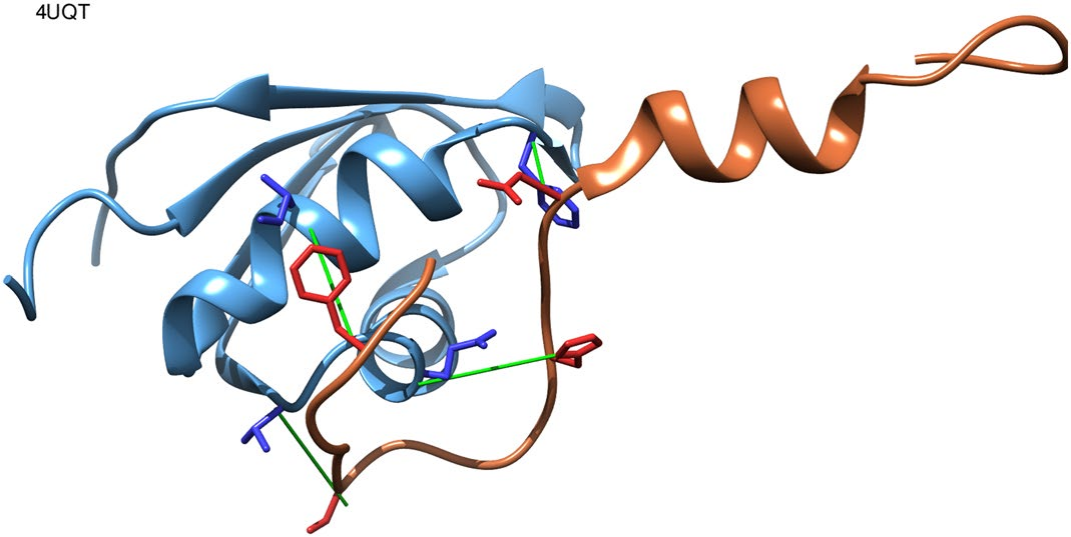
Snu17 (light blue) and Bud13 (light red) complex with high affinity (low Kd) and coevolution (pdb:4uqt). Contacting residue pairs with high covariation scores (~ 1%) are shown as sticks with green rods connecting their Cα atoms. The image was made using Chimera 1.14.1^36^.

As an example, Fig. 4 shows the complex between the ordered domain U2 SNRNP component IST3 (snu17) and its disordered partner, the pre-mRNA splicing factor CWC26 (Bud13), from the retention and splicing complex (RES) of *Saccharomyces cerevisiae*. This complex has a high AUC for inter-protein contact prediction (translated as coevolution) and a low Kd. Snu17 binds Bud13 through a large interface, in which Bud13 adopts a U-like conformation interacting with two helices of Snu17^37^. Four of the contacting residues between these two proteins are among the top 20 covarying pairs in our analysis, including two pairs involving residues close to the conserved Trp232 of bud13, which is known to be important for binding^37^.

### MFIB compactness analysis

A general observation about structures in the MFIB database is that complexes formed by coupled folding upon binding of two or more disordered chains often form a complex resembling a known globular domain. If this is the case, inter-protein coevolution of mutual folds are expected to approximate intra-protein coevolution of single-chain globular domains; this is actually what we observed in the previous section (Fig. 1 and Supplementary Figure S4). We offer additional evidence that MFIB complexes are similar to globular domains, by analyzing their radii of gyration (Rg). The distribution of Rg of MFIB complexes (inter- and intra-protein contacts) is similar to the control set of PDB monomers and differs from the control set of PDB dimers (see Supplementary Figure S7).

## Discussion

In this study, we present the first comprehensive analysis of coevolution between residues of intrinsically disordered proteins in complexes. We evaluated the performance of intra- and inter-molecular contact prediction as a proxy for coevolution in a benchmark of three established covariation methods and a naive predictor. The analysis was carried out on two manually curated datasets of IDP/IDR complexes that are already accepted in the community.

Our systematic evaluation of inter-protein residue covariation shows that the signal is weak in interactions between disordered and ordered chains, as well as between two disordered chains. However, we have seen a difference in the interaction between a disordered region and a globular domain (DIBS database) and the interaction between the two disordered chains upon binding to each other rendering a globular-like domain (MFIB database). In fact, complexes of the latter category are more similar to intra-molecular interactions observed in globular domains.

It was reassuring to be able to validate our hypothesis that the coevolutionary footprint becomes more prominent in those proteins that have a greater affinity and larger interface, laying the groundwork to develop methods to determine how stable or transient an interaction is.

We note that there are several potential causes of the weak signal observed, such as the accelerated evolutionary rate of disordered proteins, their binding promiscuity, and structural heterogeneity, dynamics and instability of their complexes.

For the future, we suggest performing an upscaled version of this study with algorithms parameterized to work with disordered proteins as soon as a larger (or different type of) dataset of IDP complexes becomes available. The results we provide can be enhanced in the future when technical limitations are mitigated.

Our analysis might have eliminated some of the mentioned technical biases but not the obstacles of the limited number of available IDP complexes (287 interactions) and the controversial reliability of the covariation calculation methods. Moreover, we should also emphasize that the sequence-alignment algorithms and homology search tools for the identification of orthologous complexes have been trained solely on globular domains and might not ideally fit with approaching disordered proteins. As the experimental techniques of structural biology for IDPs are becoming more advanced, it is expected that in the near future a significant growth of the number of available IDP complexes will be achieved. Furthermore, specific tools to optimally align IDPs will be developed, and may as well be used and made available by Pfam or other databases in the forthcoming years. As a result of all these advances, we may expect significant improvements in the covariation analysis of IDPs/IDRs.

## Materials and methods

### Dataset

#### Disordered Binding Site database (DIBS) description

DIBS is a database of 773 complexes formed by a disordered protein or region bound to an ordered protein (URL: https://dibs.enzim.ttk.mta.hu/). Each DIBS entry has two or more chains, consisting in two in the majority of the cases (656 complexes have two chains, 96 have 3, 18 have 4 and 3 have 5 chains). To characterize the database, we measured the intramolecular contacts per residue in each partner, being more than 10 in the globular partner while less than 10 in the disorder partner (see Supplementary Figure S8).

#### Mutual Folding Induced by Binding database (MFIB) description

MFIB (URL: https://mfib.enzim.ttk.mta.hu/) is a repository for protein complexes that are formed by intrinsically disordered proteins whose tertiary structure is induced by the assembly of the complex. It has 186 entries, 98 of which are homo-, the rest are hetero-complexes with two or more chains.

### Mapping and filtering procedure for DIBS and MFIB

We mapped all heterodimeric chain pairs present in DIBS and MFIB databases to Pfam database (version 32). Complexes with one or more chains not matching with any Pfam family, were filtered out. Each of the sequences of the complexes are taken as the reference sequence.

To map proteins to Pfam domains, we used the following strategy: For each complex, we extracted Uniprot IDs and sequences. The sequences are usually small segments that are not suitable to be used to scan the Pfam database. Therefore, we extracted the full protein sequences from Uniprot and used them to query Pfam. As each query sequence might match multiple Pfam domains, we only considered those that overlap with the annotated sequence in DIBS or MFIB databases. If more than one Pfam overlapped the sequences, the one with the longest overlap was taken. The pipeline procedure can be seen in Supplementary Figure S9.

Mapping and filtering resulted in a total of 228 complexes from DIBS. We ignored homodimers and duplicated MSAs due to DIBS hereto-multimer complexes (i.e. having two identical chains interacting with another one).

MFIB has 45 heterodimers and 14 tri-, tetra- and pentameric heterocomplexes. Those complexes with more than two chains were separated into dimeric units for each contacting heteromeric chains. Disordered tails not belonging to the mutual fold were trimmed. NMR structures were truncated based on MobiDB’s annotation as mobile^38^, while X-ray floppy terminal regions not forming non-local contacts were removed. Mapping and filtering resulted in 59 heterodimeric chains from the MFIB database that we could analyze.

### MFIB compactness

Structures of MFIB complexes often appear to match those of regular protein domains. In order to compare MFIB complexes with PDB monomers and dimers, we created two sets of PDB structures as controls (respectively). We compared the radius of gyration (Rg) and compactness (Rg normalized by the length) of the two PDB control sets and MFIB protein complexes.

PDB-dimers control set: we downloaded the 90% identity clustered protein X-ray dimers with high resolution (< 2 Å), then in order to obtain a length distribution similar to MFIB proteins, we took complexes with size ≤ 450 residues. This control dataset of PDB dimers constitutes 209 complexes.

PDB-monomers control set: we downloaded the 30% identity clustered protein X-ray monomers with high resolution (< 2 Å), then in order to obtain a length distribution similar to MFIB proteins, we took complexes with size ≤ 450 residues. We ended up with 375 complex structures. We clustered at 30% due to the large number of complexes obtained when clustering at 90% identity.

### Building the paired MSA

We looked for DIBS and MFIB sequences (reference sequence) in the Pfam database (Pfam sequence should be identical to DIBS and MFIB sequence).

For each complex, we concatenated proteins from each Pfam alignment that had identical taxonomic ID (NCBI taxonomy)^12^. This way, we ensured that both proteins belong to the same organism, a minimal condition for their putative interaction. If more than one protein had the same NCBI taxid, the most similar to the reference sequence was taken. We ended up with paired MSAs for 228 complexes from DIBS and 59 complexes from MFIB for the analysis.

### Diversity of paired MSAs

It is known that coevolution methods require a great number and diverse sequences to give meaningful results. To know the diversity of the MSAs, sequences were clustered at 62% and 80% identity with Hobohm-1 clustering algorithm^39^. Supplementary Figure S10 shows the number of clusters at 62% and 80% identity. The number of DIBS MSAs clustered at 62% identity with more than 100, 200 or 300 clusters is 60, 34 and 41 respectively and 111, 72 and 107 sequence clusters at 80% identity. In the case of MFIB, we produced 59 paired MSA in total, out of which 19, 12, 0 had at least 100, 200 or 300 sequence clusters at 62% identity; and 34, 16, 15 had at least 100, 200 or 300 sequence clusters at 80% identity.

Different methods perform better at different number of sequences and diversity in the MSA: e.g.: 400 clusters at 62% identity^15^; N/L ≥ 3 sequences for CCMPRED^9^, where N is the number of sequences and L is their length—for a 100 residues protein, the MSA should have 300 sequences—or ≥ 1000 sequences less than 100% identical^26^.

We also compared the number of clusters obtained at 62 and 80% identity for both the individual MSAs (order and disorder part) and the concatenated MSAs of DIBS complexes. The purpose of doing it was to determine if the number of clusters in the paired alignments changed due to the addition of the disorder part (Supplementary Figure S11).

### Computing covariation

We calculated covariation using CCMPred^9^, GaussDCA^40^, and MIz^41^ with default parameters.

MIz is a method based on Mutual Information that captures all coupled interactions between residue pairs. CCMPred and GaussDCA methods rely on statistical models to eliminate indirect coupling. CCMPred uses a pseudo-likelihood maximization on a Potts model and GaussDCA uses a multivariate Gaussian model.

We show CCMPred results in the manuscript for simplicity as all the methods gave similar results.

To uniform residue positions in PDB, DIBS, MFIB and Pfam records, all of them were mapped to the UniProt sequence, and UniProt positions were used as indices for covariation calculations.

The number of sequences and diversity of the MSA are crucial to obtain reliable results because covariation analysis is highly sensitive to them. Therefore, we evaluated our results by clustering the sequences at different percent identities, which represent the MSA diversity (see “Results” section).

The vast majority of coevolving pairs can be assumed to be in contact with each other, so contact prediction is reasonable and commonly used to assess the performance of covariation methods. The predictive performance is evaluated in terms of the area under the receiver operating characteristic (ROC) curve (AUC)^42^. An AUC value of one indicates a perfect prediction and a value of 0.5 a random prediction. In order to build the ROC curve, the coevolution scores are sorted and labeled as positive or negative if the corresponding residue pair is in contact in the PDB structure and the true positive rate is plotted against the false positive rate.

### Naive predictor

Residues close in sequence tend to give good covariation scores but these values are considered trivial as residues contiguous or close in sequence are actually always in spatial proximity. To evaluate sequence distance bias (in other words, to subtract the effect of distance), we tested a naive predictor that scores residue pairs purely based on the distance between their positions in the query sequence. This means that it does not use any information from the MSA (that is, evolutionary information). Its scoring function has one naive assumption: local contacts always occur, the closer residues are in sequence, the closer they are in space. Contacts of residue pairs located distantly in the sequence are not attempted to be predicted. The formula of the scoring function:

$$ \begin{array}{*{20}l} {{\text{score}}({\text{i}},{\text{j}}) = 10^{{ - ({\text{abs}}({\text{i}} - {\text{j}}) - 1)}} } \hfill & {{\text{for}}\;{\text{abs}}({\text{i}} - {\text{j}}) < 16,} \hfill \\ {{\text{score}}({\text{i}},{\text{j}}) = 10^{ - 15} } \hfill & {{\text{for}}\;{\text{abs}}({\text{i}} - {\text{j}}) \ge 16,} \hfill \\ \end{array} $$

where i and j are amino acid indices, and abs means the absolute value.

Supplementary Figure S1 shows the performance of the naive predictor considering different numbers of residues as “trivial contacts”.

### Optimization of contact distance and sequence distance to consider a pair of residues truly coevolving in order to do the analysis

Evaluation of the performance of methods is highly dependent on the 3D distance cutoff applied to define a contact and on the number of residues set to consider a contact “non trivial” due to lack of sequence proximity. We assessed these parameters on the structured parts of DIBS complexes, thus intra-protein contacts of globular domains serve as a reference to evaluate the methods.

The distance between residues in the structures was calculated with MIToS Julia package 41 using default parameters. We tested several distances between residues (considering all heavy atoms and heavy atoms of the side chain only) to find a meaningful threshold to use in the covariation analysis.

We also evaluated the sequence proximity between residues to consider a pair of residues truly coevolving instead of “trivial” contacts. In Supplementary Figure S1, the AUC for contact prediction of the ordered part of DIBS in all situations is shown: residue distance from 2 to 6.05 Å and excluding local contacts from 1 to 12 as trivial contacts.

Supplementary Figure S1 shows that the naive method gives almost random predictions at a sequence distance of five residues (meaning that good predictions between closer residues is only due to their proximity) and a structural distance of 4 Å between any heavy atom.

The choice of five residues apart is a good tradeoff between eliminating the trivial contribution to the signal coming from sequence neighbors, and having enough data to obtain robust results. From here onwards in the results section we will show the predictions at 4 Å and 6.05 Å (6.05 Å for comparison as it is a common distance threshold used in several papers^4,16,26^ and disregarding the prediction between five contiguous neighbors. We did not see any difference considering only side chain atoms or all atoms in structural distance measures. All tested thresholds to arrive at the final parameters to evaluate covariation can be seen in Supplementary Figure S12.

## Supplementary information

Supplementary Information
