# Supplementary material for: Chasing coevolutionary signals in intrinsically disordered proteins complexes
Source: Sci Rep. 2020 Oct 21;10:17962. doi: 10.1038/s41598-020-74791-6 (PMC7578644; doi:10.1038/s41598-020-74791-6)
Supplement: Supplementary file 1 — Supplementary Information [file 41598_2020_74791_MOESM1_ESM.docx]

**Chasing coevolutionary signals intrinsically disordered proteins complexes. Supplementary material**

Javier Iserte^+1,^ Tamás Lázár^+,2^, Silvio C.E. Tosatto, Peter Tompa^2,3^, Cristina Marino Buslje^*1^

^+^Contributed equally

*corresponding author: [cmb@leloir.org.ar](mailto:cmb@leloir.org.ar)

**Supplementary Figure S1: performance at different distances and different definition of trivial contacts.**


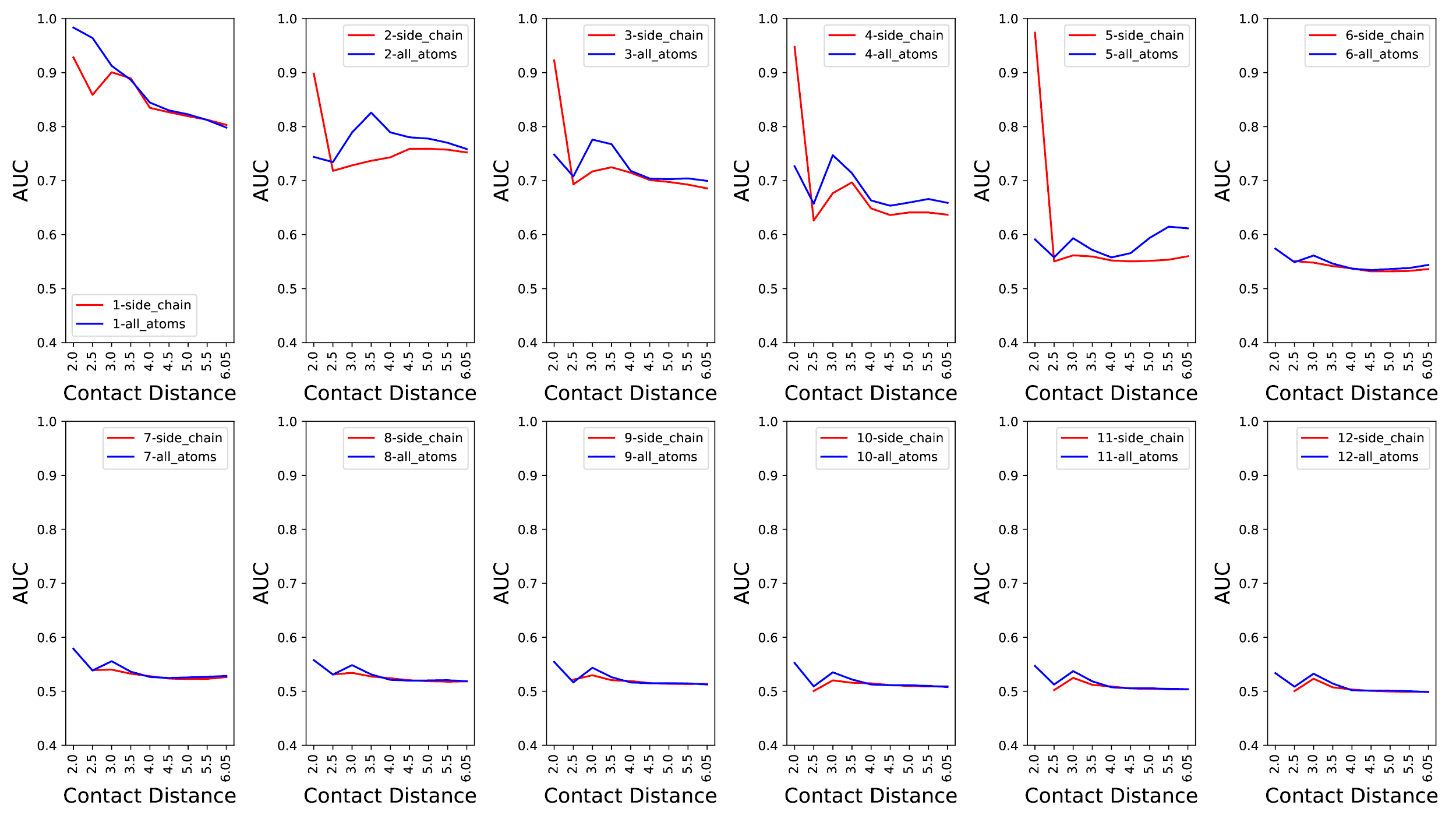


**Supplementary Figure S1:** AUC for contact prediction of the NAIVE method in the ordered part of DIBS complexes considering true positives residues located at several contact distances (from 2 to 6.05) and excluding different number of residues from the analysis, considered as “trivial contacts” instead of true positives (numbers upper right). Also, the AUC is calculated considering any heavy atoms and side chain heavy atoms in contact closer that the threshold. Shown with blue and red color lines, respectively.

**Supplementary Figure S2. Summary of covariation results on DIBS database.**

The results are shown for paired MSAs having more than 100, 200 and 300 sequence clusters, as the number of sequences in the MSA highly influences the results. We also show the results defining 5, 6, or 7 neighboring residues as trivial contacts for evaluating the unbiased prediction performance. **Supplementary Figure S2a** considers that two residues are in contact if any heavy atoms are closer than 4 and 6.05 Å, while S**upplementary Figure S2b**, considers only side-chain atoms.


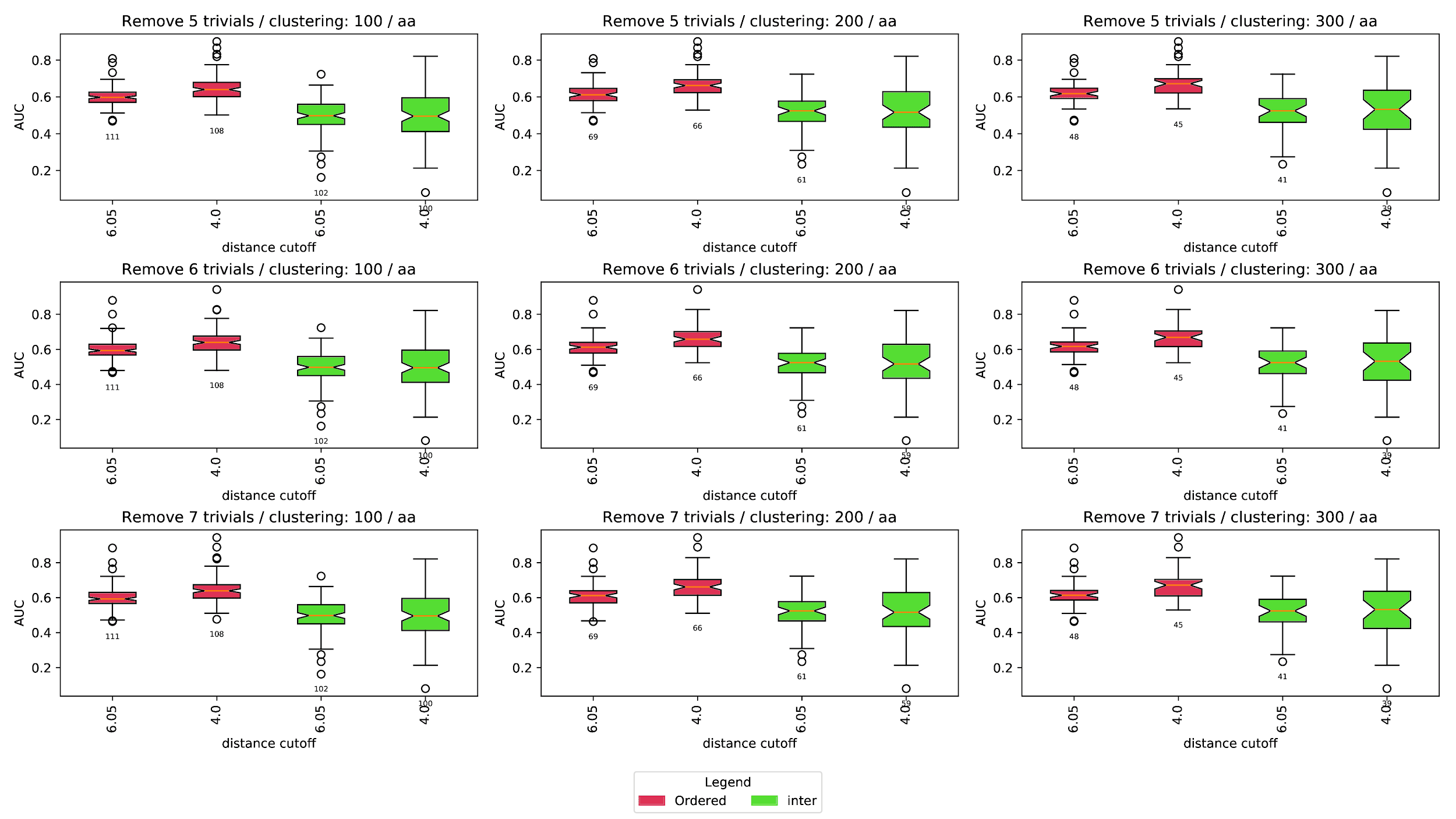


**Supplementary Figure S2a:** AUC for contact prediction. Red: intramolecular contacts, green: intermolecular contacts. Results shown at 6.05 and 4 A distance to define a contact, and excluding 5, 6 or 7 neighbours residues contacts (trivial) and at different number of sequence clusters. Contact is defined as having any heavy atom closer than 4 and 6.05 A.


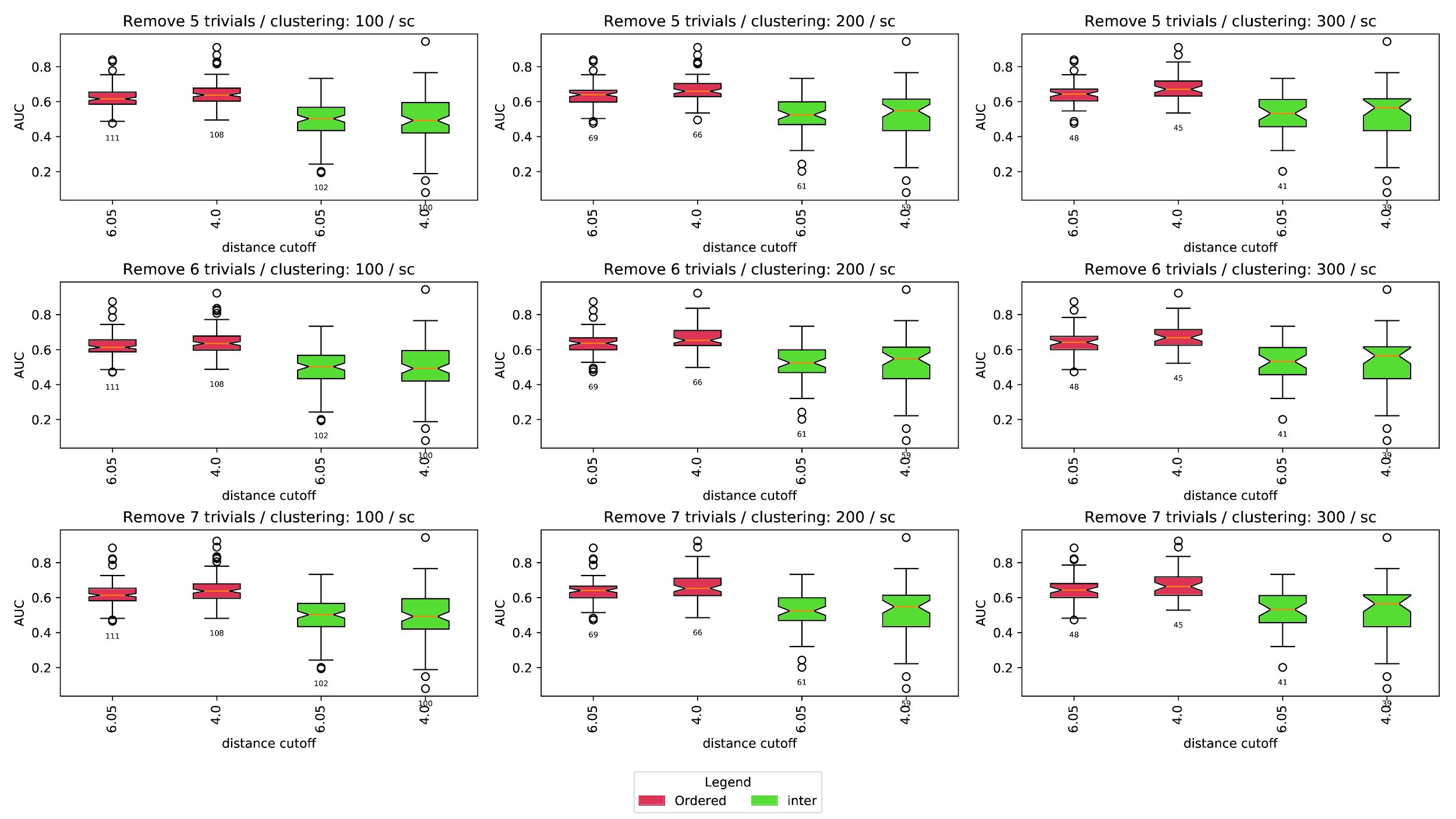


**Supplementary Figure S2b:** AUC for contact prediction. Red intramolecular contacts, green intermolecular contacts. Results shown at 6.05 and 4 A distance to define a contact, also excluding 5, 6 or 7 neighbouring residue contacts (trivial) and at different number of sequence clusters. Contact is defined as having any side chain heavy atom closer than 4 and 6.05 A.

**Supplementary Figure S3: AUC for intra DIBS protein contact prediction of the order and disorder partner and the inter protein contact prediction at several conditions.**

**A**


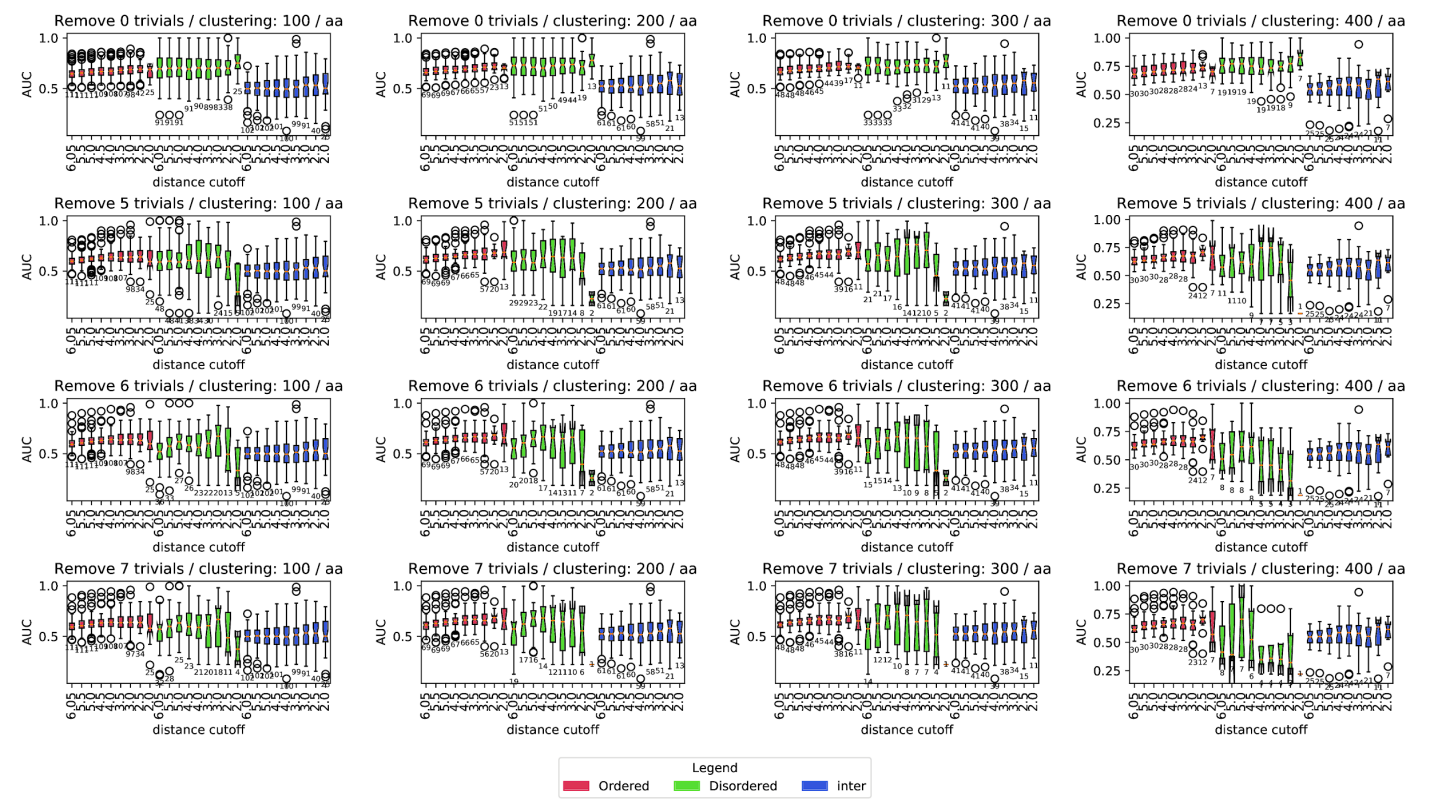


B


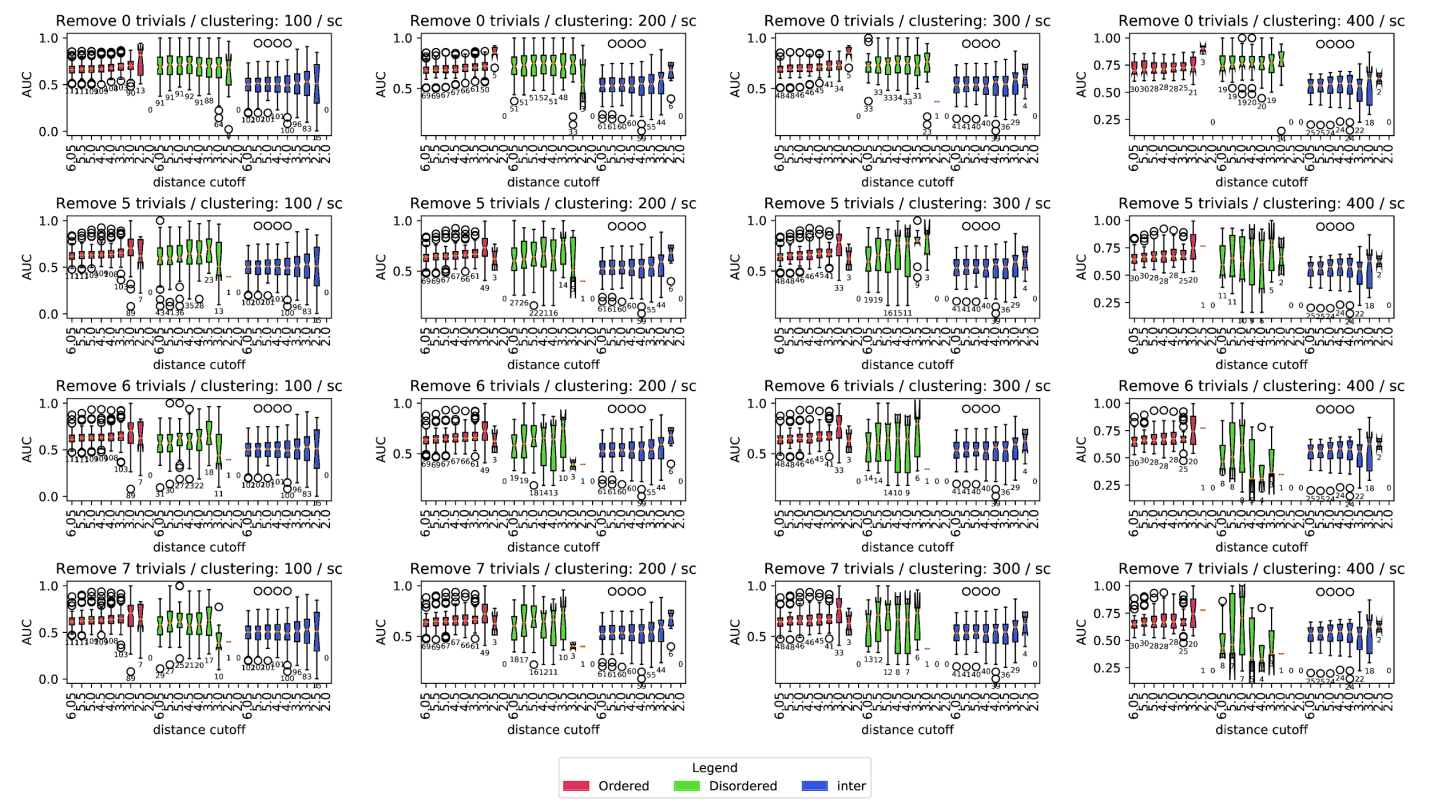


**Supplementary Figure S3:** AUC for contact prediction performance at different distance cutoffs (6.05, 5.5, 5, 4.5, 4, 3.5, 3, 2.5 A), calculated removing different number of trivials (5, 6, 7) and at different sequence clustering (>100, >200, >300). intra protein contact prediction of the ordered partner is colored red, inter protein contacts blue, and contact prediction of the disordered part is colored green.

Panel A) including all heavy atoms. Panel B) including side chain heavy atoms.

**Supplementary Figure S4: Summary of covariation results on MFIB database.**

a)


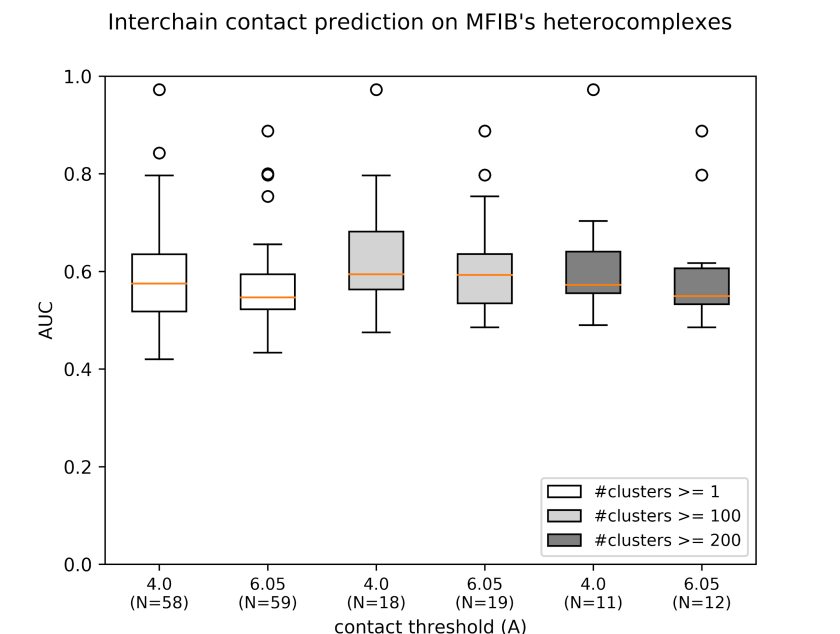


b)


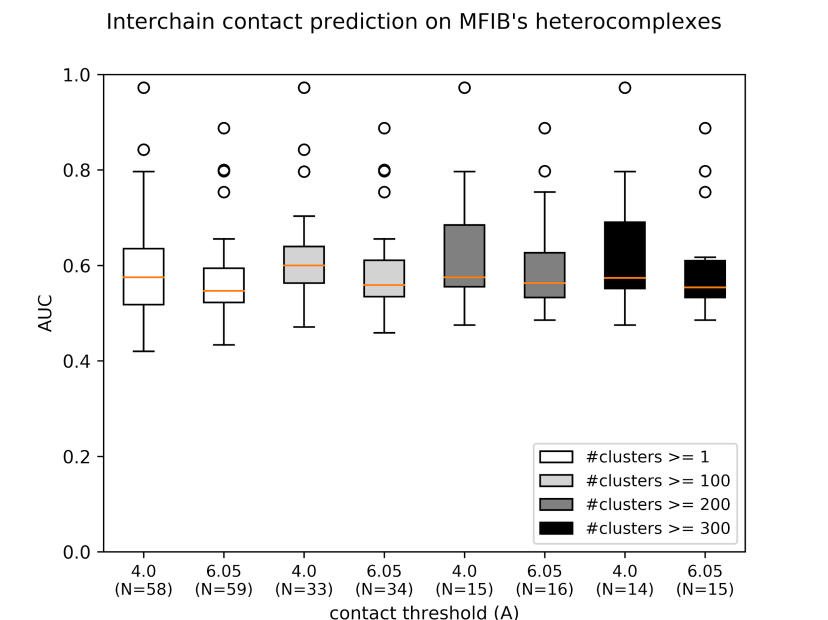


**Supplementary figure S4:** AUC for contact prediction at 4 and 6.05 A distance and different number of clusters. A) 62% clustering and B) 80% identity clustering.

**Supplementary Figure S5: Dimer resembling a single globular domain.**

**
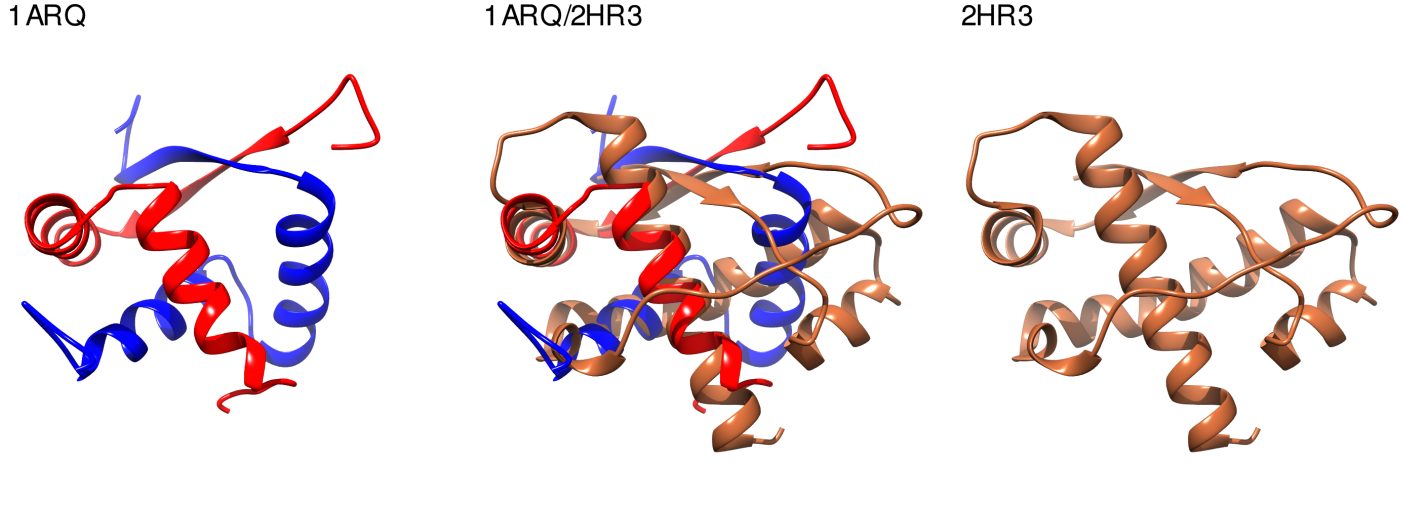
**

**Supplementary Figure S5:** Example of a dimer resembling a globular protein. One of the many heterodimer complexes that once bound make a globular domain as a single chain domain.

Left panel: chains A and B of Arc repressor PDB ID: 1arq . *Enterobacteria phage P22*; Middle panel, superposition between pdb 1arq and Dna binding protein PDB ID: 2rh3. Agrobacterium fabrum (strain C58 / ATCC 33970); right panel 2rh3. It is worth noting that 2hr3 is a single chain. The image was made using Chimera 1.14.1.

**Supplementary Figure S6: Covariation correlates with affinity and surface area.**

**a)**


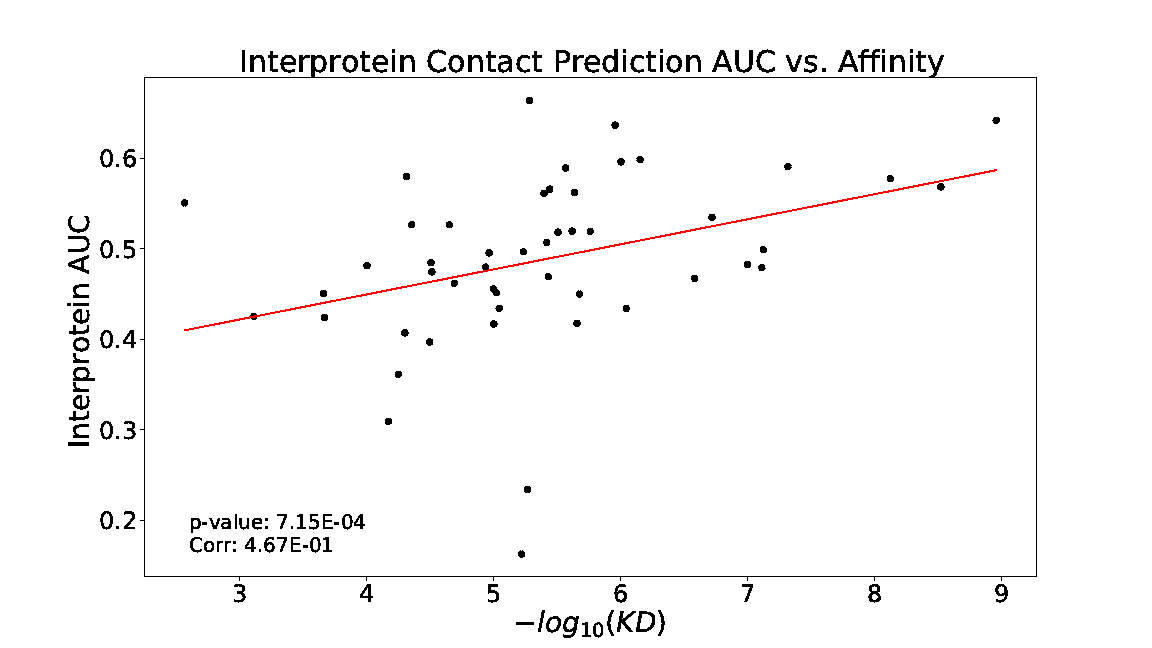


**Supplementary Figure S6a:** Correlation between Kd and AUC of inter protein contact prediction. Here we only considered protein pairs from DIBS complexes with two protein chains (pval=7.15·10^-4^, rho=0.467).

**b)**

**
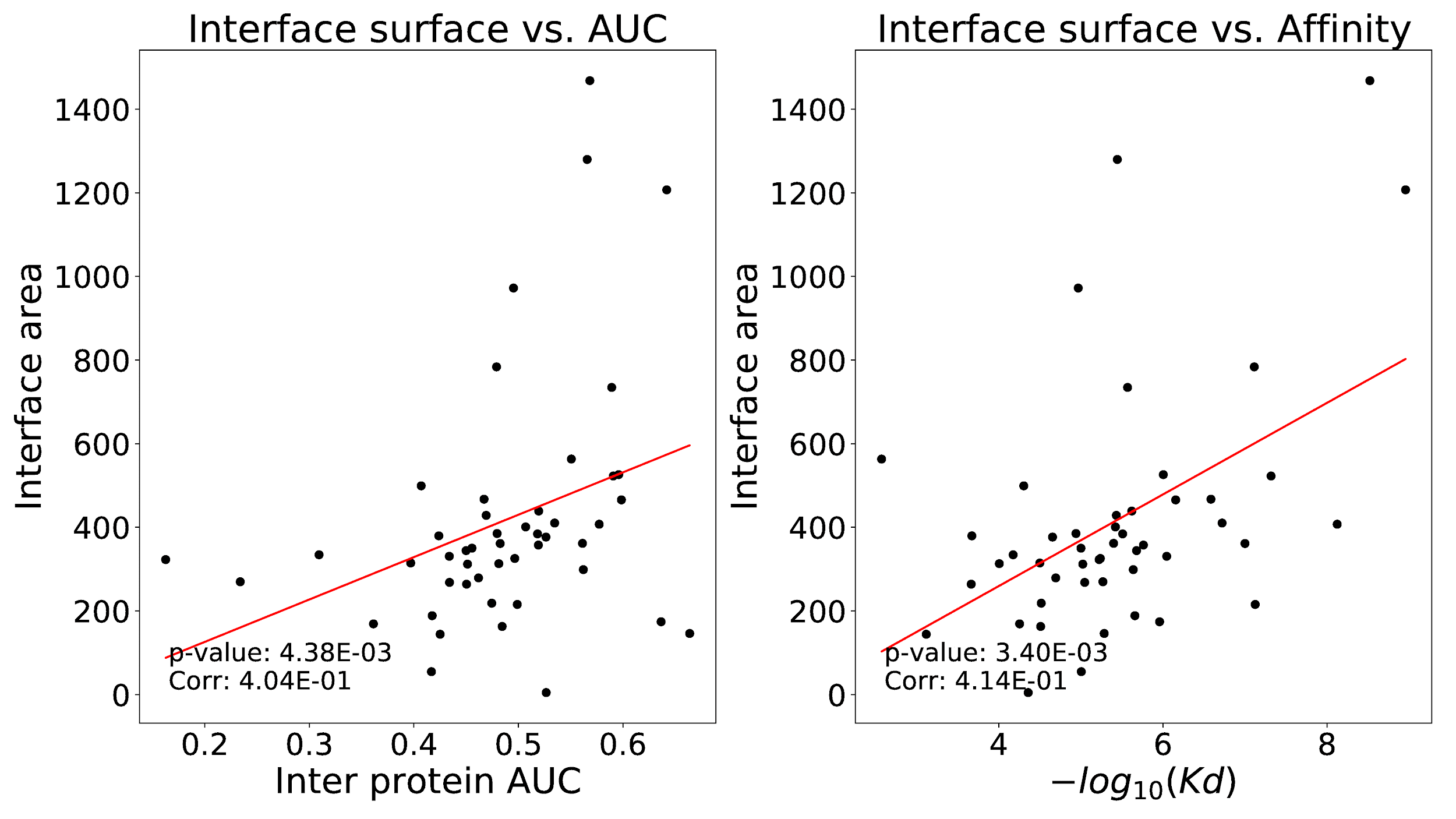
**

**Supplementary Figure S6b:** Left panel: Correlation between complex interface area and AUC for inter protein contact prediction ((pval=4.38·10^-3^, rho=0.404).); right panel: correlation between complex interface area and affinity (-log_10_(Kd)) (pval=3.40·10^-3^, rho=0.414). Here we only considered protein pairs from DIBS complexes with two protein chains

**Supplementary Figure S7: Compactness and radius of gyration on MFIB database.**

**
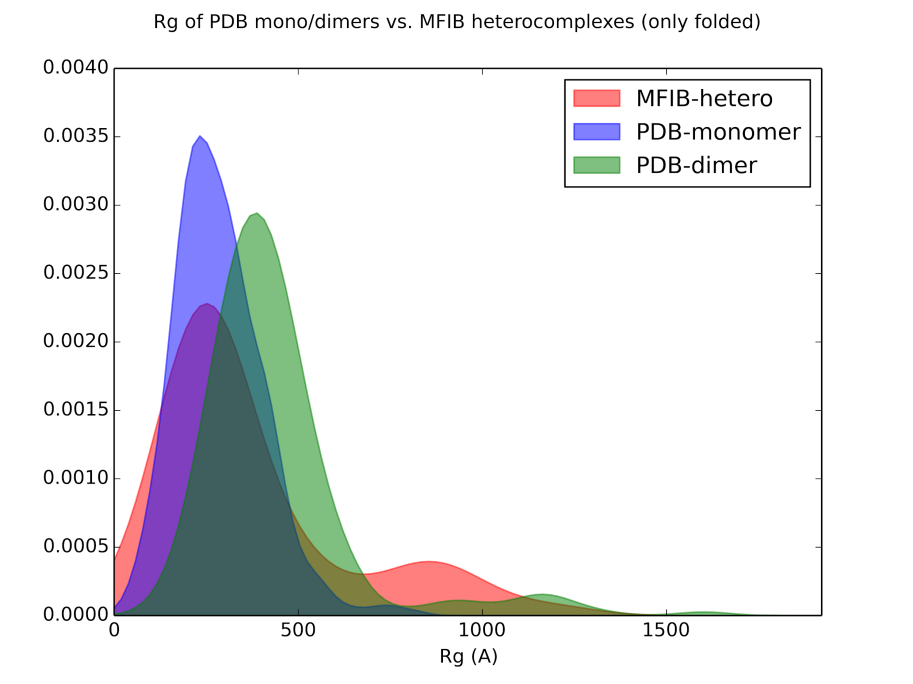
**

**
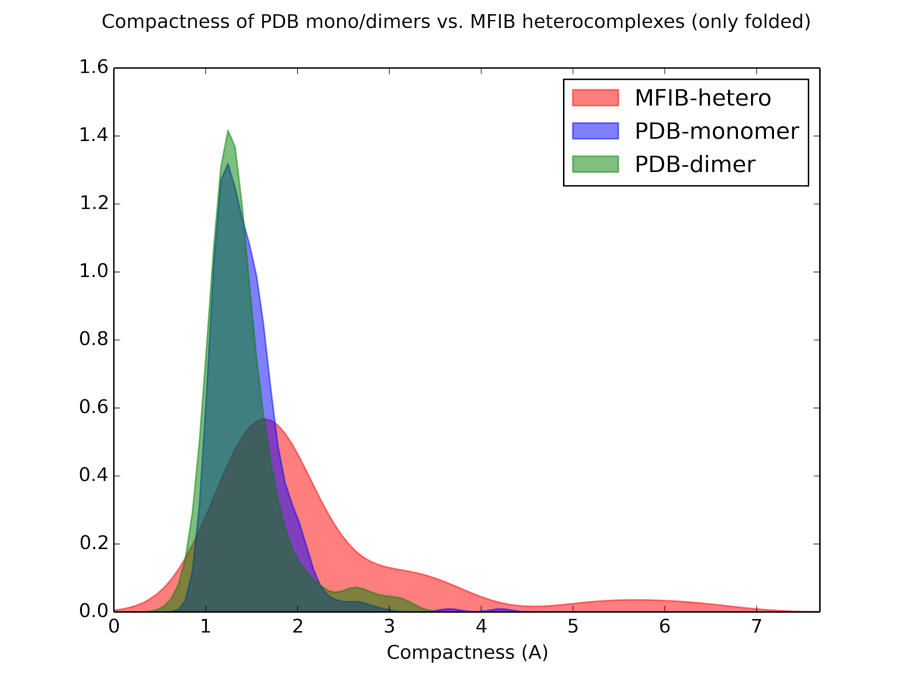
**

**Supplementary Figure S7:** Rg and compactness distributions for MFIB and the PDB control sets.

**Supplementary Figure S8: Number of intramolecular contacts of disordered and ordered proteins**


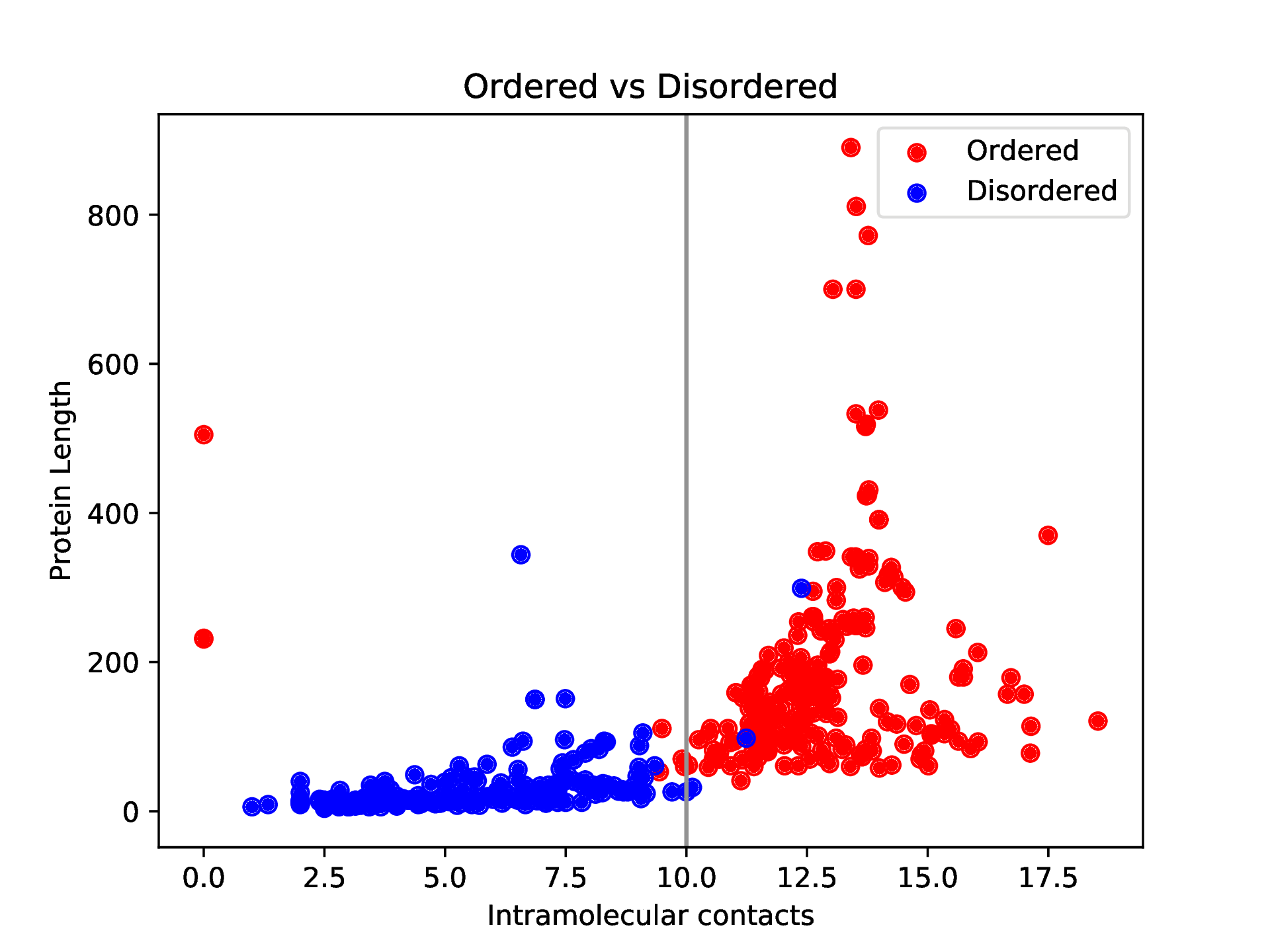


**Supplementary Figure S8**: Number of intramolecular contacts per residue in both chains. Intramolecular contacts in the globular partner of DIBS database are more than 10 while less than 10 in the disorder partner.

**Supplementary Figure S9. Pfam mapping strategy:**

**
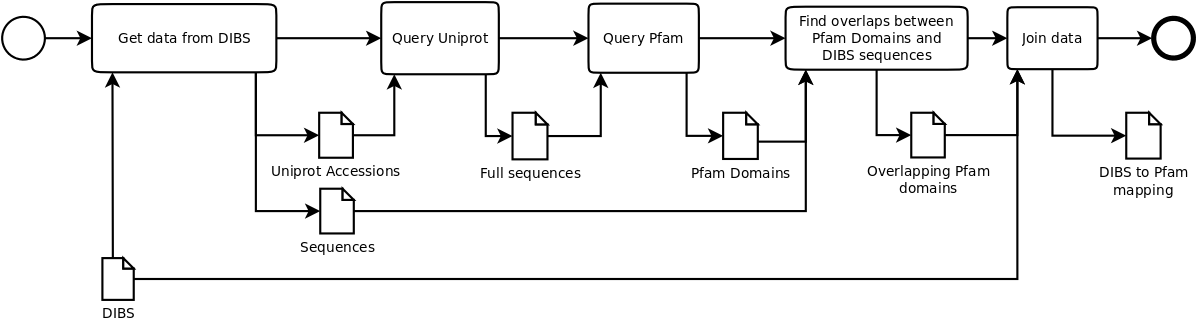
**

**Supplementary Figure S9:** Flux of Pfam mapping strategy.

**Supplementary Figure S10: Number of clusters of paired sequences in the MSA.**


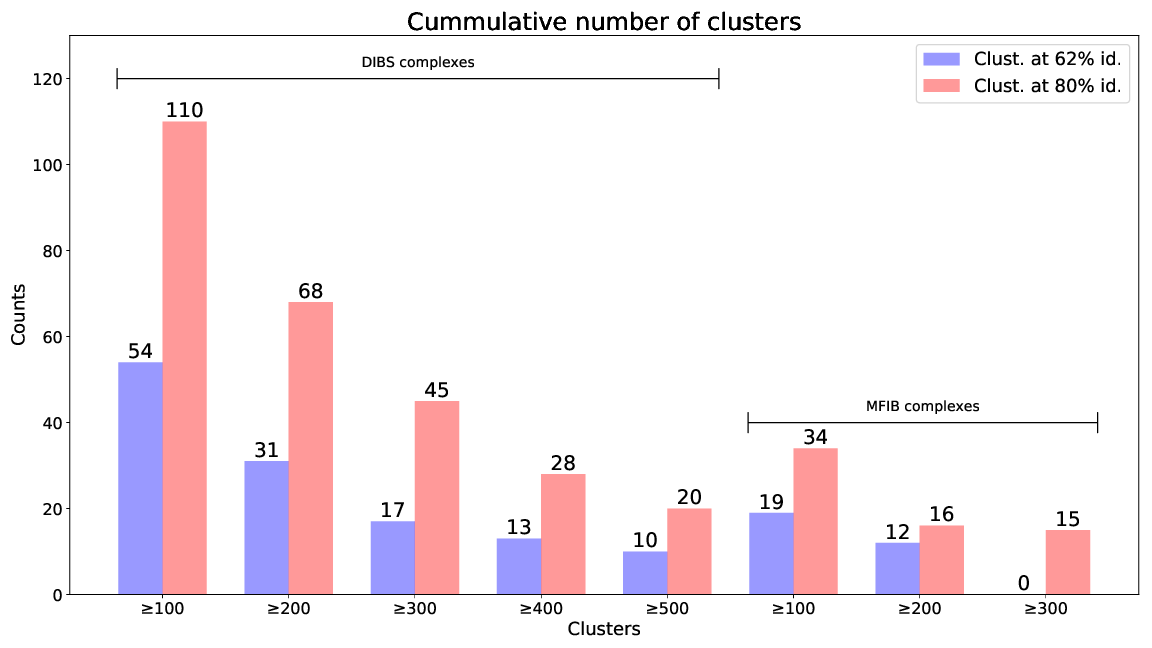


**Supplementary Figure S10:** Cumulative number of clusters at 60% and 80% identity in the MSAs for the two databases.

**Supplementary Figure S11:**


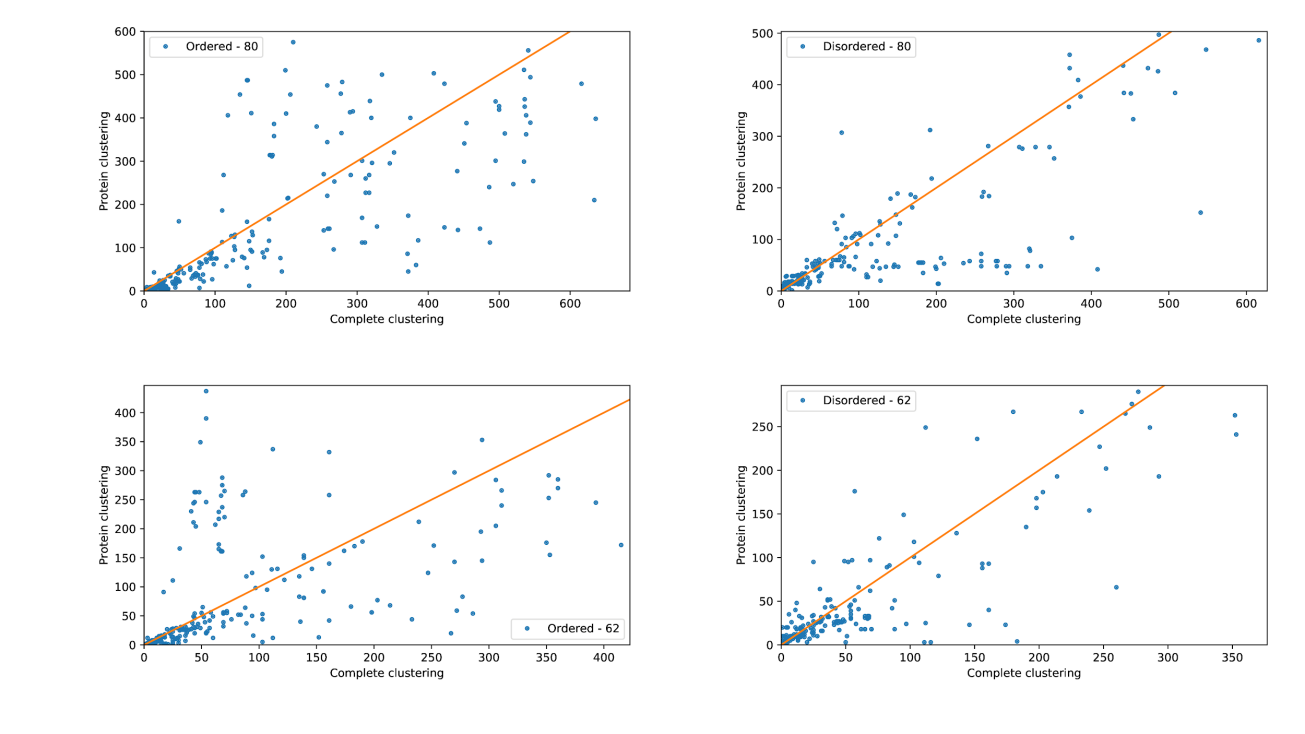


**Supplementary Figure S11:** Clustering of ordered and disordered MSAs compared with the clustering of the full (paired) MSA.

**Supplementary Figure S12:**


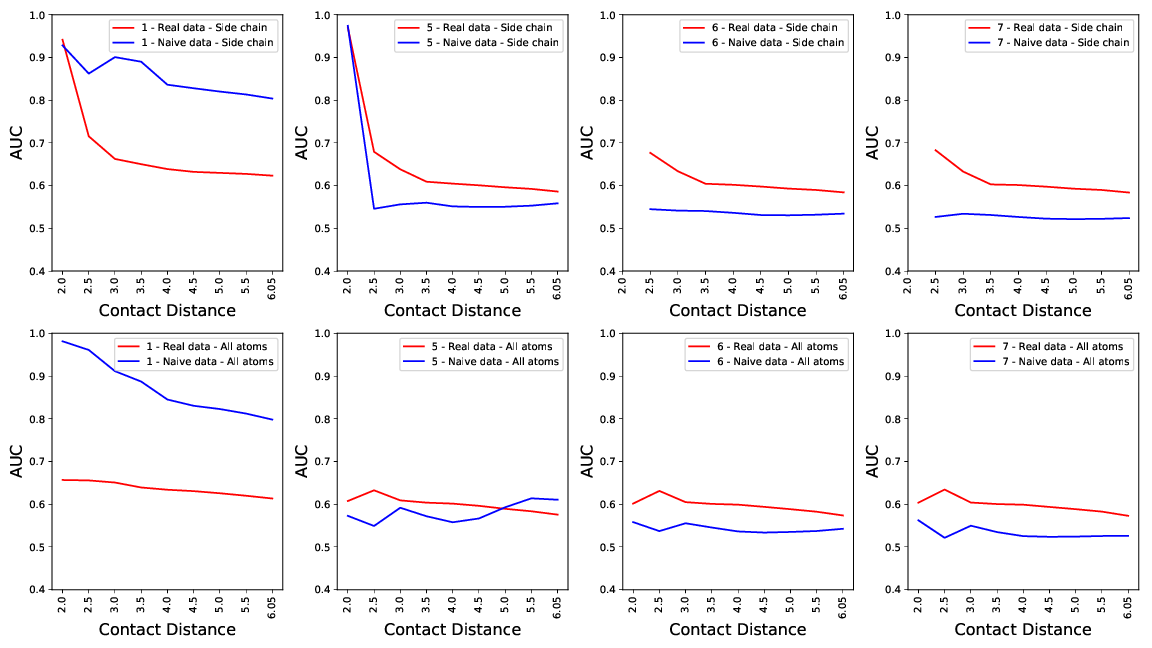


**Supplementary Figure S12:** AUC for contact prediction in the ordered part of DIBS complexes (red: real covariation method performance CCMPRED) compared with the naive predictor (blue) at several distance cutoffs and number of residues to be excluded from the analysis (“trivial contacts”), for side chain heavy atoms (upper panels) and all heavy atoms (bottom panels).

Supplementary Table S1:

| DIBS ID | Uniprot1 | Uniprot2 | AUC | log(Kd) | Interface area | PMID |
| --- | --- | --- | --- | --- | --- | --- |
| DI1000006 | O75496 | P31274 | 0.5263979496738116 | -4.653647025549361 | 376.699 | 22615398 |
| DI1000013 | P24928 | Q9H7E2 | 0.4251606978879706 | -3.1135092748275177 | 144.201 | 23066109 |
| DI1000015 | Q15637 | P26368 | 0.4989475845782639 | -7.124938736608299 | 215.568 | 12718882 |
| DI1000018 | Q71DI3 | P55201 | 0.5506499877360802 | -2.568636235841012 | 563.214 | 20400950 |
| DI1000020 | P62805 | Q92793 | 0.4504372767919912 | -3.6615435063953945 | 264.049 | 18400184 |
| DI1000022 | P68431 | Q14839 | 0.43393154224629216 | -6.045757490560675 | 330.711 | 19624289 |
| DI1000024 | Q16695 | Q96T88 | 0.4175257731958762 | -5.657577319177793 | 188.692 | 22100450 |
| DI1000030 | P62805 | Q12830 | 0.4812710437710438 | -4.00436480540245 | 313.151 | 21596426 |
| DI1000042 | O75533 | Q14498 | 0.5194444444444444 | -5.619788758288393 | 438.929 | 24795046 |
| DI1000043 | P27797 | O95166 | 0.47961383447370887 | -4.939302159646387 | 385.238 | 19154346 |
| DI1000051 | K7ES00 | Q504Y3 | 0.4167922821826952 | -5.004364805402449 | 54.7645 | 26933034 |
| DI1000053 | P68431 | Q15059 | 0.30927277642606105 | -4.1732774798310075 | 334.368 | 22464331 |
| DI1000054 | P84243 | Q96T88 | 0.5191024537236332 | -5.761953896871204 | 357.59 | 22837395 |
| DI1000061 | K7EMV3 | Q92794 | 0.49533036392372587 | -4.96657624451305 | 972.315 | 27775714 |
| DI1000075 | P24928 | Q96P16 | 0.40701754385964917 | -4.302770657240282 | 499.232 | 24997600 |
| DI1000080 | P68431 | Q03111 | 0.474512987012987 | -4.515700160653214 | 218.573 | 28241141 |
| DI1000082 | P84243 | P42568 | 0.4691732002851034 | -5.431798275933004 | 428.586 | 25417107 |
| DI1000083 | P68431 | P42568 | 0.44994553376906316 | -5.67778070526608 | 344.326 | 27105114 |
| DI1000084 | P68431 | Q9ULM3 | 0.39696333078686014 | -4.498940737782249 | 314.691 | 27103431 |
| DI1000086 | O75533 | Q96I25 | 0.6365424236748455 | -5.958607314841775 | 174.148 | 17589525 |
| DI1000111 | Q99700 | P11940 | 0.598553722929755 | -6.154901959985743 | 465.678 | 20181956 |
| DI1000125 | Q13541 | P06730 | 0.5069207189445397 | -5.42021640338319 | 400.934 | 16271312 |
| DI1000131 | P42768 | P60953 | 0.4790949038406665 | -7.113509274827517 | 783.751 | 9660763 |
| DI1000138 | O75822 | P55884 | 0.46186103458830735 | -4.6925039620867866 | 279.053 | 17190833 |
| DI1000156 | P18206 | Q9BX66 | 0.4238387978142076 | -3.669586226650809 | 379.64 | 24878663 |
| DI1000160 | P55036 | P54725 | 0.45559076233922857 | -5 | 350.132 | 12970176 |
| DI1000162 | Q15054 | Q9UBZ9 | 0.5621318564083939 | -5.638272163982407 | 298.872 | 26982350 |
| DI1000164 | Q96FZ7 | Q9UN37 | 0.496562184024267 | -5.236572006437062 | 325.531 | 18606141 |
| DI1000177 | P20645 | Q9NZ52 | 0.36131429234877505 | -4.251811972993799 | 169.119 | 11859375 |
| DI1000191 | P40855 | O75381 | 0.43411255411255406 | -5.045757490560675 | 268.142 | 19197237 |
| DI1000202 | P84243 | Q8NB78 | 0.5959886904373901 | -6.004364805402449 | 526.02 | 23260659 |
| DI1000205 | P55211 | Q13490 | 0.5907560453014998 | -7.318758762624412 | 522.668 | 19153467 |
| DI1000223 | Q13085 | P38398 | 0.6638813610358326 | -5.2839966563652 | 146.106 | 18452305 |
| DI1000244 | Q8IYD1 | P11940 | 0.518342151675485 | -5.508638306165727 | 384.297 | 14685257 |
| DI1000259 | O15047 | P61964 | 0.4671497584541063 | -6.585026652029182 | 467.316 | 22266653 |
| DI1000260 | Q9UPS6 | P61964 | 0.48260869565217385 | -7 | 361.356 | 22266653 |
| DI1000270 | P19429 | P63316 | 0.48448170300946636 | -4.508638306165727 | 162.867 | 12732641 |
| DI1100006 | P02299 | P05205 | 0.5613095238095238 | -5.397940008672037 | 361.669 | 11859155 |
| DI1100011 | P09988 | Q10103 | 0.5346867241127502 | -6.72124639904717 | 410.247 | 19362535 |
| DI1100017 | P61830 | P35189 | 0.451338199513382 | -5.0222763947111515 | 311.952 | 27089029 |
| DI1100022 | P55284 | Q99NH2 | 0.1625514403292181 | -5.221848749616356 | 322.881 | 20047332 |
| DI1100024 | P02829 | P25638 | 0.23388759104026383 | -5.267606240177031 | 269.813 | 24012479 |
| DI1100033 | P39935 | P07260 | 0.5683504455458352 | -8.522878745280337 | 1468.28 | 14675538 |
| DI1100038 | Q9JIH2 | P52293 | 0.6419223045495464 | -8.958607314841773 | 1207.16 | 16222336 |
| DI1100043 | P46947 | P40565 | 0.5773203918573808 | -8.1249387366083 | 407.334 | 25160624 |
| DI1100047 | P53112 | P80667 | 0.5266064257028114 | -4.356547323513812 | 4.72845 | 12453410 |
| DI1100086 | P41695 | P26449 | 0.5658143939393939 | -5.443697499232712 | 1279.78 | 17227844 |
| DI1100087 | P47074 | P26449 | 0.5892831945816283 | -5.568636235841012 | 734.571 | 17227844 |
